# Buprenorphine involvement in opioid overdose deaths: A retrospective analysis of postmortem toxicology in Marion County, Indiana, 2015–2021

**DOI:** 10.1016/j.dadr.2023.100131

**Published:** 2023-01-04

**Authors:** Brandon del Pozo, Danielle Atkins, Barbara Andraka-Christou, Rachel Wightman, M H Clark, Philip Huynh, Bradley Ray

**Affiliations:** a The Warren Alpert Medical School of Brown University, Providence, RI, USA; b Rhode Island Hospital, Lifespan Corporation, Providence, RI, USA; c Askew School of Public Administration, Florida State University, US; d School of Global Health Management and Informatics, University of Central Florida, Orlando, FL, USA; e Center for Behavioral Health and Justice, Wayne State University, Detroit, MI, USA; f RTI International, Research Triangle Park, NC, USA

**Keywords:** Opioid overdose, Buprenorphine, Suboxone, Polysubstance use, Fentanyl, Heroin, Harm reduction, X-waiver, DATA waiver, MOUD

## Abstract

**Background:**

Amidst an unprecedented overdose epidemic, the opioid partial agonist buprenorphine is a medication for opioid use disorder associated with reductions in overdose. Despite its efficacy, buprenorphine prescribing remains closely regulated, owing to concerns about misuse, and its possible role in overdoses.

**Methods:**

A retrospective analysis of the Marion County, Indiana coroner’s postmortem toxicology data for unintentional opioid-involved overdose deaths from 2015 through 2021. The county was chosen as a novel setting whose corner provided comprehensive overdose data. It contains Indianapolis, a large city in the US Midwest The 2,369 opioid-involved overdoses were analyzed for the presence of buprenorphine and its metabolite, as wel as potent substances associated with illicit drug use and overdose.

**Results:**

Of the 2,369 postmortem toxicology records analyzed, 55 (2.3%) indicated presence of buprenorphine. Of buprenorphine-involved cases, 51 (92.7%) involved other potent substances such as fentanyl, heroin, cocaine, methadone, and amphetamines; 4 (7.3%) were attributed to buprenorphine and liver failure, diabetic ketoacidosis, or relatively less potent substances. Fentanyl was present in 28 cases (50.9%), benzodiazepines were present in 24 (43.6%). Black opioid decedents were considerably less likely to have buprenorphine in their toxicology than White decedents.

**Conclusions:**

Buprenorphine was rarely detected in the postmortem toxicology of unintentional opioid overdoses in a major US city in the Midwest. In nearly all cases it was accompanied by other potent substances that more frequently cause fatal overdoses on their own. This study confirms findings from other geographic settings that the overdose mortality risks associated with buprenorphine are low.

## 1. Introduction

The drug overdose epidemic in the United States, driven by illicit fentanyl with the increasing involvement of stimulants, is claiming more lives than ever (Ciccarone, 2021; Kallingal & Fox, 2020; NCHS, 2022). Three medications are federally approved for treatment of opioid use disorder (OUD): methadone, buprenorphine, and naltrexone. International and domestic studies have shown that of these medications, methadone and buprenorphine have the strongest evidence base, with both consistently associated with reductions in overdose mortality and the sequelae of OUD (Santo et al., 2021; Sordo et al., 2017; Wakeman et al., 2020). Mathematical models indicate their global uptake would signifciantly reduce not only overdose death, but deaths from HIV as a consequence of injection drug use (Degenhardt et al., 2019). As compared to methadone, buprenorphine has a safer profile owing to its pharmacological ceiling on respiratory depression (Bell et al., 2009; Marteau et al., 2015; Dahan, et al., 2006), and it is available in office-based clinician settings.

In the United States, buprenorphine remains highly regulated in comparison to other Schedule III controlled substances, with federal law requiring prescribing clinicians to first obtain a waiver and then adhere to patient limits. As an opioid itself, concerns about the safety of illicitly diverted buprenorphine have prevented more relaxed policies toward the medication (Andraka-Christou, 2016). Nevertheless, studies have shown that illicit buprenorphine is primarily used for self-treatment or withdrawal prevention (Cicero et al., 2018; Silverstein et al., 2020), often when formal treatment is unavailable (Carroll et al., 2018), or to preserve a person’s sense of autonomy while meeting their self-perceived treatment needs (Hayes et al., 2021). Use of diverted buprenorphine has been associated with a reduction in unintentional overdoses (Carlson et al., 2020), and people who screen positive for non-prescribed buprenorphine before engaging in formal treatment are retained on average twice as long as those who have not (Williams et al., 2022). These findings accord with results seen in other countries that promoted buprenorphine prescribing as part of a population-level overdose reduction strategy; for example, during a concerted, national effort to increase linkage to the medication in France, it was associated with a 79% decrease in fatal opioid overdoses (Auriacombe et al., 2004; Fatseas & Auriacombe, 2007; Khazan, 2018). while domestically, in Baltimore, such an effort was accompanied by a 66% reduction (Schwartz et al., 2013).

Some U.S. jurisdictions have responded to this evidence by decriminalizing buprenorphine’s illicit possession. In 2021, Vermont and Rhode Island decriminalized the possession of non-prescribed buprenorphine (Landen, 2021; Paris, 2021), after *de facto* decriminalization by officials in Burlington, Vermont, and Philadelphia in 2018 (del Pozo, 2021;del Pozo et al., 2020). New York State is considering a bill to decriminalize the medication as well (“An act relating to decriminalizing the possession of buprenorphine,” 2022), but concerns about buprenorphine’s safety persist (Andraka-Christou, 2016; Nolan, 2019), and people who possess it illicitly remain subject to arrest in 48 states.

To inform these debates, it is critical to understand buprenorphine’s involvement in overdose deaths, the most serious potential concern related to loosening buprenorphine prescribing laws. Prior studies suggest buprenorphine-involved overdose deaths are rare, and typically involve co-exposure to other potent substances. Paone et al. (2015) analyzed 98 consecutive drug overdose cases in New York City and determined 2 (2.0%) involved buprenorphine. Public health officials in Tennessee analyzed death records from 2014 to 2018 and determined in any given year, between 3.4% and 4.7% of fatal overdoses involved a buprenorphine exposure (2020 Buprenorphine Report, 2020). Wightman et al. (2021) reviewed the postmortem toxicology of 534 opioid-involved overdose deaths (OIDs) recorded in Rhode Island from 2016 to 2018 and determined 29 (5.4%) involved buprenorphine.

Owing to relatively small sample sizes and a limited number of geographic settings, these prior studies preclude confident generalizability. Therefore, our objective was to examine the role of buprenorphine in fatal overdose in a large metropolitan area, over the course of several years. We focused on Marion County, Indiana, home to Indianapolis (2021 pop. 971,102), the 15^th^ largest city in the US, and second largest in the Midwest. We chose Marion County for the study because it was a large, geographically novel setting with hundreds of fatal overdoses a year, and the county corner had provided the study team with several years of detailed postmortem toxicology and death certificate data. We used the county coroner’s data from January 2015 through December 2021, a period of seven years, to describe: 1) the proportion of OIDs that include buprenorphine; 2) the demographics of people with and without buprenorphine-involved deaths; and 3) other substances of interest in the toxicology of opioid-involved overdose deaths.

## 2. Methods

The data for the study are from Marion County, 2015–2021. These data are drawn from a combination of sources, including the Marion County Coroner’s Office (MCCO), death certificate data, and the coroner’s toxicology reports. There is one coroner in Marion County, with jurisdiction over cases where death is the result of injury or violence; of a suspicious, unusual, or unnatural manner; or when someone in apparent good health is found dead. Accidental overdoses meet these criteria. Death certificates captured sociodemographic variables, while toxicology reports used blood and urine analyses to determine an analyte’s presence in the decedent by relying on a detection threshold established by the designated testing agency. Reporting criteria were consistent with recommended guidelines from the National Association of Medical Examiners (Davis et al., 2020) for the smallest concentration that can be reported for a given analyte, a level that may not have contributed to death, but was nonetheless detected in the decedent’s system (NMS Labs, 2022).

For the present study, we use data on opioids from county toxicology reports including buprenorphine, 6-monoacetylmorphine, morphine, codeine oxycodone, hydrocodone, oxymorphone, hydromorphone, di-hydrocodeine, norcodeine, tramadol, nortramadol, oDesmethyltramadol, methadone, EDDP, tapentadol, methadone, fentanyl and its metabolites, and U477. An inherent limitation of toxicology results is the inability to detect 6-MAM in a heroin-related overdose because of rapid transformation into natural opioids (principally morphine). Therefore, in this study as in others, cases containing morphine *and* codeine without 6-MAM were counted as heroin cases (Harruff et al., 2015; Ray et al., 2017). Drug category definitions for other substances in the analysis (e.g., prescription opioids) are provided in the supplementary materials.

Records prior to January of 2015 were excluded from this study, as the contracted labs conducting toxicology for the coroner began testing for buprenorphine and its metabolite at that time. Of the remaining 2,951 unintentional overdose death records, 2,475 (83.9%) detected any type of opioid in the decedent. Of these, 101 were excluded where toxicology was conducted by the Indiana State Department of Toxicology, which does not perform comprehensive tests, or where the testing laboratory was unknown or omitted, leaving uncertainty about whether samples were tested for buprenorphine. After excluding 5 additional cases of children under 14, the remaining 2,369 records comprised the analytic sample for the study. None of the excluded records noted buprenorphine involvement.

The records were then analyzed for detection of buprenorphine, its metabolite norbuprenorphine, and a range of other psychoactive substances and metabolites (see supplementary materials for a complete list). Coroner case narratives, which were obtained from 2016 onward, were also analyzed for mention of suspected heroin, needles, and drug paraphernalia. While these mentions do not confirm the use of a particular drug at the time of overdose, they do suggest it occurred in a setting where other drugs were present and/or being consumed. The study was deemed exempt by the Lifespan Corporation and Wayne State University IRBs because it did not involve living human subjects.

## 3. Results

### 3.1. Buprenorphine exposures

Of the toxicology reports for the 2,369 unintentional OIDs in the sample, 55 (2.3%) indicated the presence of buprenorphine or nor-buprenorphine in the decedent. While Black decedents comprised 23.7% of OIDs, they comprised 5.5% of buprenorphine involvements (n = 3). In contrast, White decedents comprised 73.3% of OIDS and 94.5% of buprenorphine-involved deaths (n = 52). No buprenorphine involvement was found among individuals of other races, and none were detected in the 14–24 year old age group. Men were more likely to have buprenorphine involvement than women, at 56.4% and 43.6%, respectively. See Table 1.

### 3.2. Polysubstance buprenorphine overdoses

Of the 55 OIDs with a buprenorphine exposure, all but 4 (n = 51, 92.7%) were polysubstance exposures to an array of drugs commonly associated with overdose on their own. In the 55 OIDs involving buprenorphine, the most common substances detected in addition to buprenorphine were fentanyl, its analogs, and their metabolites (n = 28, 50.9%), followed by benzodiazepines (*n* = 24, 43.6%). Methamphetamine (n = 21, 38.2%) and amphetamine (n = 23, 41.8%) were notably more common in buprenorphine-involved deaths than in non-buprenorphine OIDs (21.2% and 25.1%, respectively). Methadone was present in only 3.6% (n = 2) of the 55 cases. Among buprenorphine-involved deaths from 2016 onward (n = 50), we found that 20 (40%) of the case narratives mentioned drug paraphernalia was present at the scene, while 12 (24%) mentioned needles or suspected heroin. See Table 2.

In 4 cases (7.3%), buprenorphine was detected without the presence of other potent substances commonly associated with fatal overdose. Encephalopathy was listed as the cause in one case, resulting from buprenorphine’s ability to exacerbate hepatic encephalopathy by reducing liver function, a possibility for people whose liver function has been compromised (NIDDKD, 2012). In another, the coroner noted acute diabetic ketoacidosis as a contributing cause. In the third case, hydroxyzine (an antihistamine and anti-anxiety medication) was also detected, and in the fourth, antidepressants were present.

## 4. Discussion

Consistent with findings from other studies (2020 Buprenorphine Report, 2020; Paone et al., 2015; Wightman et al., 2021), buprenorphine was detected in a very low proportion of fatal OIDs in Marion County, Indiana over a seven-year period. Nearly all buprenorphine-involved OIDs in our sample (92.7%) included exposure to additional potent substances. Furthermore, the frequency of needles, paraphernalia, and suspected heroin noted at overdose scenes suggests buprenorphine was consumed in settings where the consumption of other illicit substances was likely. Together, these findings suggest buprenorphine did not play a significant role in the 2,369 fatal opioid overdoses studied.

Our study also provides useful information about co-exposures found in buprenorphine-involved deaths. The most commonly co-occurring substance was fentanyl, the primary cause of fatal overdoses in the country (Ciccarone, 2021). Since fentanyl is much more potent than buprenorphine, which has a ceiling on respiratory depression (Dahan, et al., 2006), it is likely that fentanyl was the primary driver of overdose in deaths when it was present with buprenorphine. It is also notable that benzodiazepines were detected in 43.6% of buprenorphine-involved deaths vs. 28.1% of non-buprenorphine OIDs, highlighting the concern that benzodiazepines can amplify the effects of buprenorphine by overcoming its ceiling on respiratory depression, thereby increasing the risk of overdose through co-use.

The frequent co-exposure of methamphetamine, amphetamine, and buprenorphine deserves further study, since polysubstance use has a negative impact on buprenorphine treatment retention (Tsui et al., 2020). More information is also needed about the motivations of people who use buprenorphine along with stimulants, which could improve care for people with polysubstance use disorders.

Importantly, the analysis concluded that disproportionately fewer Black decedents had buprenorphine-involved deaths as compared to OIDs generally. This finding affirms evidence of inequitable access to buprenorphine at a time when the risk of overdose in communities of color is becoming more disparate and acute (Friedman et al., 2022; Hansen et al., 2016; Wu et al., 2016), with growth in access to this treatment medication primarily accruing to predominantly White communities (Schuler et al., 2021). The findings here are also congruent with evidence that Black Medicaid recipients were more likely to be prescribed buprenorphine for shorter than optimal durations, at less effective doses, than their White counterparts (Landis et al., 2022), and that national increases in buprenorphine prescribing were primarily driven by White patients with the ability to pay cash for the medication (Lagisetty et al., 2019). These consistent disparities, combined with the concern that Black people who had resolved a significant alcohol or drug problem were more likely to hold negative attitudes toward buprenorphine (Bergman et al., 2020), suggest it is critical expand the availability and acceptability of buprenorphine in Black communities. Pharmacy-based buprenorphine prescribing/dispensing holds particular promise in this regard (Coon et al., 2020; Peckham et al., 2021), as do primary care physicians (Wakeman & Barnett, 2018), both of which would provide Black communities with access points that are more numerous, carry less stigma, and more likely to accept public and private insurance.

In summary, we found that over the course of seven years in one of the nation’s large metropolitan areas, buprenorphine rarely appeared in the postmortem toxicology of 2,369 opioid-involved overdose deaths. With the exception of 4 cases, the highly potent co-occurring substances detected in these decedents preclude us from ascribing a significant role to buprenorphine when it did appear. In the 4 cases where these potent substances were not detected, either complications of diabetes or diminished liver function contributed to death, or buprenorphine’s distinct role remained unclear. Our results confirm the need to reassess the safety risks of buprenorphine to inform policy discussions about its regulation, and the decriminalization of its illicit possession (Messinger et al., 2022). If concerns about buprenorphine’s role in fatal overdose are a key reason for restrictive laws and regulations, our study’s findings do not support this rationale.

### 4.1. Limitations

This study has limitations. We cannot exclude the possibility that the low number of buprenorphine-involved OIDs in the sample is the result of a low volume of buprenorphine prescribing, since the study did not examine prescribing data. As noted, the study excluded records where the lab was unknown, or where there was cause to believe the tests were not comprehensive, to be confident that the analytic sample contained only lab results that reliably tested for buprenorphine. Since none of these excluded cases involved buprenorphine, however, it is possible that the actual rate of buprenorphine exposure was lower than we observed in our ultimate sample.

We are also limited in our ability to draw conclusions using data extracted from coroner case narratives about the presence of heroin and drug paraphernalia. Although the presence of these items corroborates toxicology results about co-exposure to other potent substances, the items are not a definitive indication that a decedent consumed a particular substance during the incident that led to a fatal overdose. The narratives are based on preliminary investigations at the scene, descriptions such as “heroin” were not confirmed by lab tests, and drug paraphernalia can be used to consume a range of substances. At most, the narratives provide evidence that a decedent was found in a location that also contained drugs believed to be heroin or the items used to consume illicit substances. Moreover, our study does not illustrate the cause of overdoses, a complex endeavor when buprenorphine is involved (Bishop-Freeman et al., 2021); it can only show what substances were present in a decedent postmortem. Nevertheless, considerable variation in the potency of the substances detected allows for hypotheses about their relative roles in an OID (e.g., buprenorphine vs. fentanyl). Finally, the study sample is drawn from one metropolitan area; that area, however, includes the city of Indianapolis, which is among the largest cities in the United States. Our findings are consistent with those discussed above in Tennessee, New York City, and Rhode Island, contributing to the growing evidence for geographic generalization of buprenorphine’s safety.

## 5. Conclusion

To our knowledge, this is the largest U.S. toxicology study to date examining buprenorphine-involved overdose deaths, and the first in the Midwest region. Our findings confirm prior studies that buprenorphine is rarely present in, nor a significant cause of, OIDs, and speak to the potential for broad generalizability. These results suggest placing a greater emphasis on expanding buprenorphine’s accessibility through elimination of the federal waiver requirement and patient limits, even if doing so may increase diversion. Additionally, our findings suggest an increasing frequency of co-occurring buprenorphine and stimulant use, highlighting the need for effective treatment for people with polysubstance use disorders.

## Supplementary Material

Supplementary Material

## Figures and Tables

**Table 1:** Demographic data for a sample of 2,369 accidental opioid-involved overdose deaths in Marion County, Indiana, January 2015 – December 2021

	Buprenorphine-involved overdose deaths^b^ (n = 55)		Non-buprenorphine-involved opioid overdose deaths (n = 2,314)		Total opioid-involved overdose deaths (n = 2,369)	
Age	Number	Percent	Number	Percent	Number	Percent

14–24	-	0.0	201	8.7	201	8.5
25–34	17	30.9	736	31.8	753	31.8
35–54	29	52.7	1,051	45.4	1,078	45.5
55+	9	16.4	326	14.1	334	14.1
**Sex**	**Number**	**Percent**	**Number**	**Percent**	**Number**	**Percent**
Male	31	56.4	1,564	67.6	1,594	67.3
Female	24	43.6	750	32.4	775	32.7
**Race** ^a^	**Number**	**Percent**	**Number**	**Percent**	**Number**	**Percent**
Black	3	5.5	557	24.1	560	23.7
White	52	94.5	1,679	72.8	1,731	73.3
Asian	-	0.0	5	0.2	5	0.2
Other	-	0.0	12	0.5	12	0.5
Hispanic	-	0.0	54	2.3	54	2.3

a Hispanic ethnicity was recorded in the coroner’s data as a race variable precluding other selections. Due to missing data for race in 7 cases, total sample size for those variables is 2,362.

b See supplementary data for drug category definitions.

**Table 2:** Commonly-consumed recreational/illicit substances detected in the postmortem toxicology of a sample of 2,369 accidental opioid-involved overdose deaths in Marion County, Indiana, January 2015 – December 2021

	Buprenorphine-involved overdoses ^a^ (n = 55)		Non-buprenorphine-involved opioid overdoses (n = 2,314)		Total opioid-involved overdoses (n = 2,369)	
Substance	Number	Percent	Number	Percent	Number	Percent

Fentanyl	28	50.9	1,756	75.9	1784	75.3
Benzodiazepine	24	43.6	650	28.1	675	28.5
Amphetamine	23	41.8	581	25.1	604	25.5
Methamphetamine	21	38.2	491	21.2	512	21.6
Prescription opioids	16	29.1	771	33.3	787	33.2
Cannabinoids	16	29.1	535	23.1	550	23.2
Ethanol	13	23.6	463	20.0	476	20.1
Cocaine	10	18.2	574	24.8	585	24.7
Heroin	9	16.4	548	23.7	557	23.5
Gabapentin	5	9.1	148	6.4	154	6.5
Methadone	2	3.6	81	3.5	83	3.5
**Coroner case narrative mentions of evidence present near decedent at scene of overdose, 2016 – 2021** ^b^						
Paraphernalia	24	40.0	903	43.0	925	42.9
Needles/“heroin”	13	20.0	503	23.9	515	23.9
Total cases	50		2,106		2,156	

Note: Individual overdose deaths could involve exposure to multiple substances.

a See supplementary materials for a list of substances tested on toxicology and drug category definitions.

b Extracted from coroner reports for 2,156 opioid-involved overdoses in Marion County, Indiana, January 2016 - December 2021. In case narratives, one incident may have both categories mentioned in the narrative. The notation of suspected heroin in narratives does not reflect confirmatory testing.
