# Supplementary material for: Buprenorphine involvement in opioid overdose deaths: A retrospective analysis of postmortem toxicology in Marion County, Indiana, 2015-2021
Source: Drug Alcohol Depend Rep. 2023 Jan 4;6:100131. doi: 10.1016/j.dadr.2023.100131 (PMC9910498; doi:10.1016/j.dadr.2023.100131)
Supplement: Supplementary file 1 [file mmc1.docx]

**Supplementary Material – Substance category definitions**

- **Buprenorphine** was defined as buprenorphine and/or norbuprenorphine.
- **Opioids** were defined as: 6-monoacetylmorphine, morphine, codeine oxycodone, hydrocodone, oxymorphone, hydromorphone, dihydrocodeine, norcodeine, tramadol, nortramadol, oDesmethyltramadol, methadone, EDDP, tapentadol, methadone, fentanyl and its metabolites, and U477.
- **Prescription opioid-involved deaths** were defined as those from: morphine, codeine oxycodone, hydrocodone, oxymorphone, hydromorphone, dihydrocodeine, norcodeine, tramadol, nortramadol, oDesmethyltramadol, methadone, EDDP, and tapentadol.norcodeine, tramadol, nortramadol, oDesmethyltramadol, methadone, EDDP, and tapentadol.
- **Heroin** was defined as 6- monoacetylmorphine *or* cases with both morphine *and* codeine, but not 6-monoacetylmorphine since heroin quickly metabolizes to morphine or codeine, which can indicate a heroin overdose in toxicology data. We did not include cases with both morphine *and* codeine *and* a prescription opioid in the heroin measure to avoid double-counting of these deaths. This approach is consisten with prior research (Harruff, Couper, & Banta-Green, 2015; Ray et al., 2017).

**Supplementary Material – List of substances to be detected by postmortem toxicology performed for the Marion County, Indiana Coroner’s Office during the period of analysis (substance category definitions follow this list)**

| Alcohols |
| --- |
| Ethanol |
| Acetone |
| Isopropanol |
| Methanol |
| Chloroethane |
| Amphetamines |
| Amphetamine |
| Methamphetamine |
| Ephedrine |
| MDMA |
| MDA |
| Phenylpropanolamine |
| Phentermine |
| Analgesics |
| Acetaminophen |
| Salicylates |
| Naproxen |
| Phenacetin |
| Antibiotics |
| Azithromycin |
| Piperacillin |
| Levofloxacin |
| Anticonvulsants |
| Gabapentin |
| Oxcarbazepine |
| Topiramate |
| Lamotrigine |
| Levetiracetam |
| Pregabalin |
| Zonisamide |
| Valproicacid |
| Primidone |
| Antidepressants |
| Paroxetine |
| Fluoxetine |
| Norfluoxetine |
| Mirtazapine |
| Citalopram |
| Trazodone |
| Chlorophenylpiperazine |
| Doxepin |
| Nordoxepin |
| Fluvoxamine |
| Sertraline |
| Norsertraline |
| Desmethylsertraline |
| Amitriptyline |
| Nortriptyline |
| Duloxetine |
| Norvenlafaxine |
| Bupropion |
| Hydroxybupriopion |
| Venlafaxine |
| ODesmethylvenlafaxine |
| mCPP |
| Desmethyldoxepin |
| Atomoxetine |
| Antihistamines |
| Hydroxyzine |
| Diphenhydramine |
| Doxylamine |
| Chloropheniramine |
| Guaifenesin |
| Antipsycotics |
| Clozapine |
| Norclozapine |
| Quetiapine |
| Amlodipine |
| Aripiprazole |
| Olanzapine |
| Buspirone |
| Benzatropine |
| Risperidone |
| 9_Hydroxyrisperidone |
| Phenytoin |
| Ziprasidone |
| Barbituates |
| Phenobarbital |
| Butalbital |
| Benzodiazepine |
| Lorazepam |
| Clonazepam |
| Nordiazapam |
| Diazepam |
| Alprazolam |
| 7AminoClonazepam |
| aOHAlprazolam |
| Temazepam |
| Oxazepam |
| Chlordiazepoxide |
| AlphaHydroxyalprazolam |
| Demoxepam |
| Midazolam |
| Flubramazolam |
| Phenazepam |
| Delorazepam |
| Diclazepam |
| Hydroxytriazolam |
| Etizolam |
| Cardiovascular |
| Verapamil |
| Metoprolol |
| Diltiazem |
| Propranolol |
| Hydrochlorothiazide |
| Lidocaine |
| Clonidine |
| Gastrointestionals |
| Promethazine |
| Dicyclomine |
| Loperamide |
| Desmethylloperamide |
| Illicits |
| 6_MAM |
| Heroin_from_combo |
| Cocaine |
| Benzoylecgonine |
| Cocaethylene |
| PCP (Pencyclidine) |
| Ketamine |
| Norketamine |
| Cathinone |
| Cannibinoids |
| THC |
| THC_COOH |
| Carboxy_THC |
| Delta_9_THC |
| Delta_9_Carboxy_THC |
| 11_Hydroxy_Delta_9_THC |
| Synthetic_Cannibinods |
| 5F_ADB_THC_synthetic |
| 4-fluoro-MDMB-BINACA |
| 5-fluoro-MDMB-PICA |
| Rx_Opioids |
| Morphine |
| Codeine |
| Fentanyl |
| Norfentanyl |
| Carfentanil |
| Oxycodone |
| Hydrocodone |
| Oxymorphone |
| Hydromorphone |
| Dihydrocodeine |
| Norcodeine |
| Tramadol |
| Nortramadol |
| o-Desmethyltramadol |
| Methadone |
| EDDP |
| Partial_Opiod_Agonist |
| Buprenorphine |
| Norbuprenorphine |
| Normeperidine |
| Designer_Opioids |
| Acetyl_Fentanyl |
| Acryl_Fentanyl |
| 4_ANPP |
| Para_Fluorobutyryl_Fentanyl |
| Methoxyacetyl_Fentanyl |
| Furanyl_Fetanyl |
| Butyryfentanyl |
| Valeryl_Fentanyl |
| U477 |
| Opioid_Antagonist |
| Naloxone |
| Miscellaneous |
| Hydroxychloroquine |
| Atenolol |
| Narcotics |
| Glucose |
| Carboxyhemoglobin |
| Psychoactivesubstances |
| Difluoroethane |
| Amiodarone |
| Yohimbine |
| Gilipizide |
| Sildenafil |
| Quinine |
| Levamisole |
| n_desmethylsildenafil |
| 6_beta_naltrexol |
| Xylazine |
| Trihexphenidyl |
| Furosemide |
| Tadalafil |
| Theophylline |
| Trihexyphenidyl |
| Warfarin |
| Mitragynine |
| Muscle_Relaxants |
| Methocarbamol |
| Tizanadine |
| Cyclobenzaprine |
| Carisoprodol |
| Meprobamate |
| Orphenadrine |
| Neurologicals |
| Benztropine |
| OTC_Cold_Remedies |
| Dextromethorphan |
| Pseudoephedrine |
| Norpseudoephedrine |
| Sedatives_Hypnotics |
| Eszopiclone |
| Zolpidem |
